# Gastroprotective potential of fatty compounds from *Persea americana* seed: an experimental report

**DOI:** 10.1007/s10787-026-02419-8

**Published:** 2026-10-08

**Authors:** Greice Rafaele Alves, Benhur Judah Cury, Bruna Longo, Alexandre Bella-Cruz, Raphael Gilioli Heineck, Rafaela Carvalho Faustino, Yasmim Damian Pompeo Benevides, Luisa Mota da Silva, Rivaldo Niero

**Affiliations:** 1 Graduate Program in Pharmaceutical Sciences, University of Vale do Itajaí (UNIVALI), Rua Uruguai 458, Itajaí, SC 88.302.901 Brazil; 2 Multiuser Analytical Instrumentation Laboratory, University of Vale do Itajaí (UNIVALI), Rua Uruguai 458, SC 88.302.901 Itajaí, Brazil; 3 Chemical Pharmaceutical Research Center (NIQFAR), University of Vale do Itajaí (UNIVALI), Rua Uruguai 458, Itajaí, SC Brazil; 4 Department of Pharmacology, Graduate Program in Pharmacology, Federal University of Santa Catarina, Florianópolis, SC Brazil

**Keywords:** *Persea americana*, Gastroprotection, Gastric ulcer

## Abstract

*Persea americana* (Lauraceae) is a fruit widely cultivated in tropical and subtropical regions, commonly known as the avocado. Although the seeds represent a considerable portion of the whole fruit, scientific research on their phytochemistry and biological effects is still in its early stages. This study investigated the gastroprotective effect of the crude methanolic extract (CME-PA; 30–300 mg/kg), the fractions hexane (HF-PA; 100–1000 mg/kg), dichloromethane (DCMF-PA; 30–1000 mg/kg), and ethyl acetate (EAF-PA; 1000 mg/kg) at doses proportional to their yields, as well as isolated compounds (**1**–**4**) and a mixture of compounds (**M**). Gastroprotection was evaluated using the acidified ethanol-induced ulcer model. In addition, the study assessed histological, oxidative, and inflammatory parameters in the gastric mucosa, as well as the in vitro anti-*H. pylori* activity. Although CME-PA did not prevent the onset of gastric lesions, the HF-PA (300 and 1000 mg/kg), DCMF-PA (1000 mg/kg), and EAF-PA (1000 mg/kg) fractions attenuated acidified ethanol–induced injury by 56.73%, 74.93%, 80.53%, and 52.91%, respectively. Similarly, compounds **4** (300 mg/kg) and **M** (1, 3, and 10 mg/kg) reduced lesion formation by 52.16%, 56.02%, 66.90%, and 72.89%. Furthermore, compounds **4** (300 mg/kg) and **M** (3 mg/kg) increased PAS-stained mucin content. Notably, the mixture **M**, at all tested doses, enhanced the antioxidant defenses of the gastric mucosa and reduced inflammation. Despite these findings, the extract, fractions, and isolated compounds did not exhibit anti-*H. pylori* activity. Overall, this study provides promising phytochemical and pharmacological evidence, given the significant gastroprotective effects of fractions and isolated compounds against acidified ethanol–induced gastric ulcers. These results highlight the potential of *P. americana* seeds, often regarded as a by-product of the fruit, as a valuable natural resource for the prevention of gastric ulcer disease.

**Supplementary Information:**

The online version contains supplementary material available at https://doi.org/10.1007/s10787-026-02419-8.

## Introduction

Avocados (*Persea americana* Mill., Lauraceae family) are nutrient-dense fruits that are eaten as fresh pulp or processed pulp by the industry to make packed slices, dehydrated avocado, oil extraction, puree, or guacamole (Jimenez et al. 2020). Avocado peels and seeds are by-products generated from both fresh consumption and industrial avocado processing, with seeds being the majority, since they comprise up to 30% of the fruit’s weight, resulting in waste and environmental contamination (Alissa et al. 2020).

Despite the effectiveness of the drugs to treat gastric ulcers, most of them have several adverse effects, mainly in long-term treatment (Yuan et al. 2006). The residual bioactive substances in food by-products can therefore be a creative source in this field, and avocado by-products can be introduced into sustainable markets and a circular economy strategy according to the various activities attributed to avocado seed components (Colombo and Papetti 2019; Nascimento et al. 2025), which include a high concentration of fatty alcohols and acids, carotenoids, phenolic compounds, and alkanols (Dabas et al. 2013; Kosińska et al. 2012).

While previous studies have demonstrated the gastroprotective benefits of the avocado seed ethyl acetate fraction rich in phenolics (Athaydes et al. 2019), the potential of other, less polar components of avocado seeds remains underexplored. Given the diversity of bioactive compounds in avocado seeds, this study aimed to investigate the gastroprotective potential of low-polarity fractions (hexane and dichloromethane) from *P. americana* seeds, exploring the hypothesis that, in addition to phenolic compounds, other chemical components in these fractions may contribute to antiulcer effects, thereby expanding the understanding of avocado seeds as a source of bioactive compounds with therapeutic potential.

Avocado contains ubiquitous lipid families that are present in other plant matrices; however, it has unique fatty acid (FA) derivatives such as fatty alcohol esters (FAEs) and polyhydroxylated fatty alcohols (PFAs) (Rodríguez-Sánchez et al. 2025). Avocado FAE and PFA have been associated with different and relevant bioactivities, including plant defense (Néeman et al. 1970; Domergue et al. 2000), health benefits (Rodriguez-Sánchez et al., 2015; Tcheng et al. 2017; Ahmed et al. 2019; Bhuyan et al. 2019), and antimicrobial activity (Wu et al. 2019; Rodriguez-Sánchez et al., 2015). In addition, previous studies have also reported that avocado-derived polyhydroxylated fatty alcohols (PFAs), such as avocadene and avocadyne, exhibit mitochondrial antioxidant properties (Tcheng et al. 2022) and potential anti-*Helicobacter pylori* activity (Le et al., 2025), which could potentially contribute to the protection of the gastric mucosa against ulcerogenic agents.

Furthermore, the therapeutic or preventative role of certain fatty alcohols, such as those found in plant extracts, has been documented in the literature (Montserrat-de la Paz et al. 2014; Fernández-Arche et al. 2009), and a Phase I human clinical trial confirmed the activity and safety of polyhydroxylated fatty alcohols (Ahmed et al. 2019). In addition, avocadene and avocadyne also possess unique physicochemical properties that facilitate absorption and biodistribution after oral intake (Ahmed et al. 2020, 2025). Therefore, given the significant potential of PFAs for human nutrition and health and the presence of fatty alcohols in the low-polarity fractions and their potential biological activities, we hypothesized that they might play a role in the observed gastroprotective effects of *P. americana* seed extracts.

## Materials and methods

### Drugs and reagents

Bovine serum albumin (BSA), 5,5′-dithiobis (2-nitrobenzoic acid) (DTNB), 3,3′,5,5′-tetramethylbenzidine (TMB), Brucella broth, glutathione, carbenoxolone, hexadecyltrimethylammonium bromide (HTAB), penicillin, pyrogallol, sodium phosphate, and thiobarbituric acid (all from Sigma, St. Louis, USA), absolute ethanol, ethyl acetate, hexane, hydrochloric acid, formaldehyde, hydrogen peroxide, magnesium chloride, methanol, sodium acetate, sodium carbonate, sucrose, trichloroacetic acid (Vetec, Rio de Janeiro, RJ, Brazil), dimethyl sulfoxide and N, N-dimethylformamide (DMSO, Synth, Diadema, SP, Brazil), and fetal bovine serum (FBS, Gibco) were among the materials used.

### Plant material

*Persea americana* Mill. seeds were collected in the town of Ribeirão D’areia-Pedras Grandes, SC, Brazil (latitude 28^°^57’26.6’’S and longitude 49^°^25’21.4’’W), in August 2020 and identified by Dr. Oscar Benigno Iza. An exsiccate (number HBR 57935) was deposited at the Barbosa Rodrigues herbarium in Itajaí, SC, Brazil.

### Extraction and isolation of compounds

**Fig. 1:**
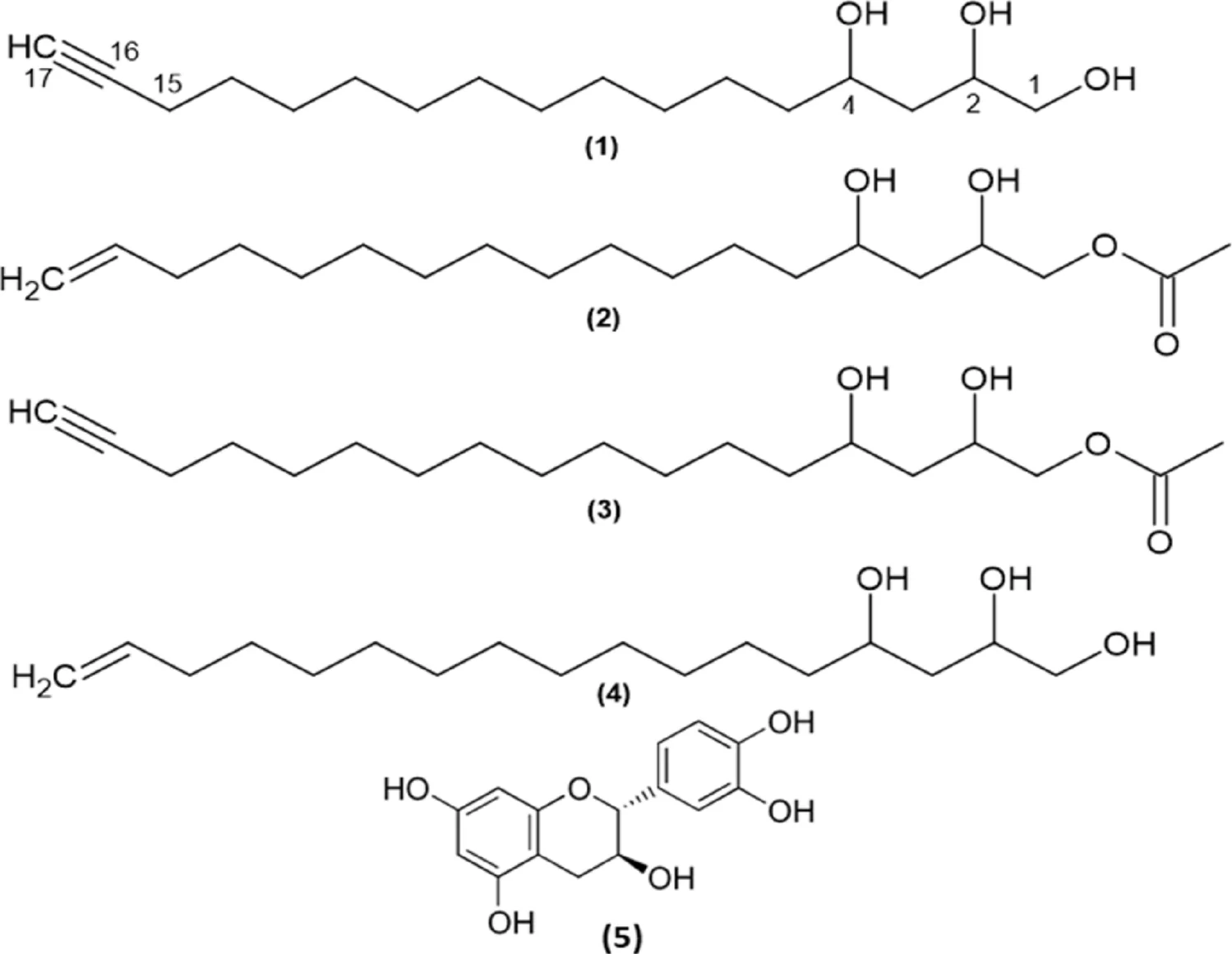
Molecular structure of isolated compounds

The extraction of the extract and isolation of compounds were carried out as described by Pereira et al. (2021) with some modifications. Briefly, 700 g of dry and ground seeds were submitted to maceration processes with methanol at room temperature for 7 days. After filtration, the solvent was removed by rotatory evaporation under reduced pressure at 50 °C. After the crude methanolic extract (CME-PA; yield 13 g), which was successively partitioned (each 300 mL x 4) with n-hexane (HF-PA; yield 5.70 g), dichloromethane (DCMF-PA; yield 0.51 g), and ethyl acetate (EAF-PA; yield 0.89 g). Subsequently, the hexane fraction was chromatographed on a silica gel column (0.04–0.063 mm, 2.5 × 50 cm) and eluted with a gradient of hexane: acetone (100→0) to give 94 fractions, grouped by similar phytochemical profiles using thin-layer chromatography (TLC). Subfraction 73–92 was rechromatographed by a similar eluant system, yielding the compounds **1–4** (Fig. 1). Anisaldehyde, sulfuric acid, and ferric chloride as reagent colors were used to visualize the spots. The purity of all compounds was initially assessed by TLC and direct comparison of peak areas in LC-ESI-MS chromatograms with values between 93 and 98%. The compounds were identified by their spectral data (FTIR, EIMS, ^1^H-NMR, and ^13^C-NMR; see Supplementary Figures S2-S5) in comparison with previously reported data (Domergue et al. 2000; Oberlies et al. 1998).

### In vitro anti-H. pylori activity

The broth dilution method, as outlined by CLSI (2003) with modifications for microaerobiosis and culture media, was used to calculate the Minimum Inhibitory Concentration (MIC) (CLSI 2003; Stenger-Moura et al. 2021; Meurer et al. 2023). The bacteria (*Helicobacter pylori*, ATCC 43629) were cultured in microaerophilia for 72 h after being activated in blood agar to create the *H. pylori* inoculum. Then, compared with the 0.5 McFarland scale, 4 to 5 colonies were separated and suspended in saline solutions. The final inoculum contained the equivalent of 10^5^ cells/mL after this suspension was diluted in fetal bovine serum (FBS) and Brucella broth.

After dissolving the positive control ampicillin in dimethyl sulfoxide (DMSO), it was diluted with the culture medium until it reached a concentration of 40 mg/mL. The growth medium, the solvent, and the bacterial inoculum made up the negative control. The extract, fractions, and isolated compounds were serially double-diluted and combined with Brucella broth supplemented with 10% FBS at a concentration of 2.5 mg/mL. This resulted in final concentrations ranging from 2500 to 5 µg/mL. Plates were incubated for 3 days in a microaerobic atmosphere at 37 °C. Following incubation, the plates were carefully examined, and the MIC for that chemical was determined by taking note of the lowest concentration that demonstrated total growth inhibition.

Three independent experiments, each carried out in technical duplicate to ensure reproducibility, were performed. The medium’s DMSO content didn’t go above 1%. The antimicrobial activity of the samples was classified according to the criteria established by Holetz et al. (2002), where MIC values below 100 µg/mL indicate strong activity, values between 100 and 625 µg/mL indicate moderate activity, values between 625 and 1250 µg/mL indicate weak activity, and values above 1250 µg/mL indicate the material is inactive.

### Animals

The Ethics Committee on the Use of Animals (CEUA) of the University of Vale do Itajaí (UNIVALI) approved all the experiments (approval number: 010/21). The study employed 60-day-old female Swiss mice from the central vivarium of UNIVALI. The animals had unrestricted access to food and water and were kept at 23 °C with a 12-hour light/dark cycle for at least seven days before experimental treatment. Animals were randomly allocated into experimental groups based on their body weight to ensure homogeneity. Due to logistical constraints, investigators were not blinded during treatment administration or endpoint evaluations.

### Acidified ethanol-induced ulcers model

The mice were fasted for 8 h with free access to water prior to the ethanol-induced ulcer procedures, as described by Meurer et al. (2023). The animals were divided into different groups (*n* = 6–8). In the first set of experiments, the mice received CME-PA (30, 100, or 300 mg/kg), carbenoxolone (200 mg/kg; positive control), or vehicle (10% DMSO, 1 mL/kg; negative control) orally. After one hour, the animals received an ethanol-acidified solution (60% ethanol/0.03 M HCl, 100 mL/10 kg, p.o.) and were euthanized one hour thereafter. Then, the stomachs were removed and opened along the greater curvature to evaluate the mucosa appearance.

In the second and third experiments, the gastroprotective effect of the fractions was evaluated, and HF-PA (100, 300, and 1000 mg/kg, p.o.), DCMF-PA (30, 100, and 1000 mg/kg, p.o.), and EAF-PA (1000 mg/kg, p.o.) were administered 1 h prior to the ethanol/HCl intake.

Fatty compounds **1–4** were isolated from HF-PA, as well as a mixture containing compounds **2** and **3** plus β-sitosterol (defined as **M**), and their gastroprotective effects were also evaluated. Therefore, in the fourth set of experiments, the mice received **1–4** and **M** in a dose calculated in accordance with their field in HF-PA and the minimum effective dose of HF-PA (2.1, 0.5, 1, 7.3, and 0.6 mg/kg, respectively, p.o.) 1 h before the ethanol/HCl administration. Compounds **1**, **2**, **3**, and **4** and **M** were obtained from HF-PA with yields of 0.70%, 0.16%, 0.33%, 2.43%, and 0.20%, respectively. The HF-PA of *P. americana* seeds was found to be effective in gastroprotection at a dose of 300 mg/kg. Consequently, to investigate the contribution of these compounds and **M** to the observed HF-PA gastroprotective effect at 300 mg/kg, they were tested at doses proportional to their yields and the effective dose of the hexane fraction. As a result, compounds 1, 2, 3, and 4 were administered at doses of 2.1 mg/kg, 0.5 mg/kg, 1 mg/kg, and 7.3 mg/kg, respectively, and **M** was tested at 0.6 mg/kg, allowing for a direct comparison of their individual contributions to the overall gastroprotective activity of the hexane fraction.

In the fifth trial, compound **4** was also isolated from the DCMF-PA majority (19.2%), and its gastroprotective action was also tested at a dose of 192 mg/kg, which was calculated in accordance with its yield regarding DCMF-PA and the minimum gastroprotective dose of DCMF-PA (1000 mg/kg).

Finally, in a fifth set of experiments, compound **4** obtained from the DCMF-PA fraction (30, 100, and 300 mg/kg, p.o.) and the mixture of compounds (**M**) obtained from the HF-PA fraction (1, 3, and 10 mg/kg, p.o.) were evaluated in a dose–response curve, since they had shown greater efficacy in protecting the gastric mucosa against acidified ethanol–induced lesions in the previous experiments.

In all experiments the images of the stomachs were captured to determine the ulcer area using EARP^®^ software (Meurer et al. 2023).

### Histological and histochemical evaluation

The protocols described by Pereira et al. (2013) were followed for the histological and histochemical analyses. Briefly, the ulcerated area was excised and fixed in a solution containing 85% ethanol, 10% formalin, and 5% acetic acid. After 24 h, the fixative solution was replaced with 70% ethanol. The samples were subsequently embedded in paraffin and sectioned at 5 μm. The sections were stained with hematoxylin and eosin (H&E), and histopathological changes were evaluated by light microscopy at 100× magnification.

In parallel, neutral mucins in the gastric mucosa were detected using periodic acid–Schiff (PAS) histochemical staining. Gastric sections were examined by light microscopy, and images were acquired at 400× magnification. For each animal (*n* = 6 per group), four non-overlapping microscopic fields from different regions of the gastric mucosa were randomly selected. Measurements obtained from these fields were averaged to generate a single value per animal. PAS-positive staining was quantified using ImageJ software. Image acquisition and quantitative analyses were performed by a single observer blinded to the experimental group allocation.

### Determination of oxidative status parameters

The oxidative status of the gastric mucosa is influenced by several oxidative markers, including reduced glutathione (GSH), malondialdehyde (MDA), catalase (CAT), glutathione-S-transferase (GST), superoxide dismutase (SOD), and antioxidant defense molecules. These markers can alter ulcerogenesis. Phosphate buffer (pH 6.5) was used to collect and homogenize ulcer site samples from each group. Malondialdehyde (MDA, Percário et al. 1994) and reduced glutathione (GSH, Sedlak and Lindsay 1968) were then measured using the resultant homogenate. The resulting homogenates were then centrifuged at 11,000 g for 20 min at 4 °C, and the supernatant’s GST and CAT activities were assessed. A thorough procedure for these analyses may be found in Da Silva et al. (2015).

### Evaluation of myeloperoxidase activity

The myeloperoxidase (MPO) activity was evaluated in the precipitation because it is an indirect indicator of neutrophil infiltration. The samples were suspended in 0.5% hexadecyltrimethylammonium bromide in PBS (pH 6.0). The suspensions were then centrifuged at 40,000 g for 15 min, and the resultant supernatant was added to H_2_O_2_ and 3,3′,5,5′-tetramethylbenzidine to determine the MPO activity at a wavelength of $\:\lambda\:$ **=** 620 nm (Bradley et al. 1982). This parameter was expressed as units of millioptical density (mO.D.)/mg of protein. Protein concentrations were determined by the Bradford method (Bio-Rad, Hercules, CA, USA), using bovine serum albumin (BSA).

### Statistical analyses

First, the collected data were initially tested using the Shapiro-Wilk normality test to determine normal distributions. Results were analyzed by one-way analysis of variance (ANOVA) followed by Bonferroni’s post hoc test and expressed as means ± standard error of the means (SEM). Values corresponding to more than 2 standard deviations in relation to the means were considered outliers. However, no biological observations or animals were excluded from the final statistical analysis (*n* = 6 animals per group). All analyses were carried out using GraphPad Prism 7.0 (GraphPad Software, San Diego, USA). A p-value < 0.05 was considered significant.

## Results

### Phytochemical profile of *P. americana* seeds

The seeds (700 g) were dried, grounded, and subjected to static maceration in methanol (MeOH) for 7 days. The methanolic extract was concentrated by rotary evaporation, yielding 13 g of crude methanolic extract (CME). Subsequently, the extract was subjected to partitioning with solvents of increasing polarity, obtaining the following semi-purified fractions: fraction hexane (HF-PA, 5.70 g), fraction dichloromethane (DCMF-PA, 0.51 g), and fraction ethyl acetate (EAF-PA, 0.89 g). The hexane fraction was subjected to open column chromatography (OCC), using silica gel as the stationary phase and eluting in a hexane: acetone gradient, yielding 94 subfractions that were grouped according to the profile observed by thin layer chromatography (TLC).

Subsequently, the sub-fractions were rechromatographed by flash chromatography (FC), using the same elution system, yielding four compounds in the form of white powdery solids and initially named HF-PA 161–169 (**1**, 39.9 mg, yield 0.70%, purity 98.32%); HF-PA 99–100 (**2**, 9.5 mg, yield 0.16%, purity 94.23%); HF-PA 108–114 (**3**, 18.8 mg, yield 0.33%, purity 95.35%); and HF-PA 137–142 (**4**, 138.5 mg, yield 2.43%, purity 97.34%). In addition, from the HF-PA fraction, we also identified a mixture of compounds, which we initially designated as HF-PA 17–18 (**M**, mixture, 11.4 mg). This mixture contains compounds **2**, **3**, **4**, and β-sitosterol in the respective mass percentages of 7.12%, 2.65%, 6.12%, and 0.27%, according to the peak area obtained directly from the Total Ion Chromatogram (see Supplementary Figure S1).

From the EAF-PA, using the same chromatographic technique and eluted with a chloroform: methanol mixture (0→100), it was possible to isolate the flavonoid catechin (**5**) (Fig. 1). Interestingly, compound **4** was also isolated from DCMF-PA, using a chloroform: methanol eluting mixture (0→100), in a majority manner, yielding 97.9 mg (19.2%). All compounds were analyzed by nuclear magnetic resonance spectroscopic techniques (NMR) of hydrogen and carbon-13 (see Supplementary Figures S2-S5) compared with literature data (Oberlies et al. 1998; Lôbo et al. 2008; Domergue et al. 2000). In addition, comparative spectral data (Tables 1 and 2) can be observed in the supplementary material.

**Table 1:** Compound **M**, but not compound **4**, isolated from *P. americana*, attenuates oxidative stress in the gastric mucosa of mice with ulcers induced by acidified ethanol

Grups	GSH	GST	CAT
Naive	344 ± 19	1.23 ± 0.09	2.15 ± 2.26
Vehicle	293 ± 04^b^	29.86 ± 2.52^d^	1.19 ± 0.30^b^
Carbenoxolone	275 ± 15^c^	5.18 ± 1.60^f^	2.52 ± 0.27^f^
4 (300 mg/kg)	288 ± 09^b^	11.64 ± 2.75^a, f^	0.61 ± 0.07^d^
M (1 mg/kg)	276 ± 03^c^	4.09 ± 1.01^f^	0.82 ± 0.08^c^
M (3 mg/kg)	271 ± 12^c^	8.54 ± 1.80^a, f^	0.67 ± 0.07^d^
M (10 mg/kg)	267 ± 04^c^	9.40 ± 1.74^b, f^	2.10 ± 0.30^e^

Reduced glutathione (GSH, µg/mg of tissue); glutathione S-transferase (GST, mmol of GSH/mg of protein); catalase (CAT, mmol H_2_O_2_/mg of protein/min). Animals were pretreated with vehicle (Veh, 1% DMSO, 1 mL/kg, p.o.), carbenoxolone (Cbx, 200 mg/kg), compound **4** (1,2,4-trihydroxy-n-heptadec-16-ene, avocadene) isolated from DCMF-PA (300 mg/kg, p.o.), or **M** (mixture of compounds) isolated from HF-PA (1, 3, and 10 mg/kg, p.o.). Results are expressed as means ± S.E.M. (*n* = 6–8). One-way ANOVA followed by Bonferroni’s test. ^a^ *p* < 0.05, ^b^ *p* < 0.01, ^c^ *p* < 0.001, ^d^ *p* < 0.0001 when compared to the naive group (non-ulcerated). ^e^ *p* < 0.01 and ^f^ *p* < 0.0001 when compared to the vehicle group

**Table 2:** Summary of treatments, doses, and outcomes of extracts, fractions, and isolated compounds from *P. americana* in the gastric mucosa of mice with acidified ethanol–induced ulcers

Treatments	Doses (mg/kg)	Results
CME-PA	30	Did not prevent acidified ethanol–induced gastric ulcers.
	100	
	300	
HF-PA	100	Did not prevent acidified ethanol–induced gastric ulcers.
	300	Significantly reduced gastric ulcers induced by acidified ethanol.
	1000	
HF-PA compounds		
1	2.1	Did not prevent acidified ethanol–induced gastric ulcers.
2	0.5	
3	1	
4	7.3	
M	0.6	Significantly reduced gastric ulcers induced by acidified ethanol.
	1	Significantly reduced gastric ulcers induced by acidified ethanol. Reduced GSH and MDA levels. Reduced GST, CAT, and MPO activity. Did not prevent PAS depletion.
	3	Significantly reduced gastric ulcers induced by acidified ethanol. Reduced GSH and MDA levels. Reduced GST, CAT, and MPO activity. Increased PAS production.
	10	Significantly reduced gastric ulcers induced by acidified ethanol. Reduced GSH and MDA levels. Reduced GST and MPO activity. Increased CAT activity. Did not prevent PAS depletion.
DCMF-PA	30	Did not prevent acidified ethanol–induced gastric ulcers.
	100	
	1000	Significantly reduced gastric ulcers induced by acidified ethanol.
DCMF-PA compound		
4	192	Significantly reduced gastric ulcers induced by acidified ethanol.
	30	Did not prevent acidified ethanol–induced gastric ulcers.
	100	
	300	Significantly reduced gastric ulcers induced by acidified ethanol. Reduced GSH levels and CAT activity. Increased MDA levels. Increased GST and MPO activity. Increased PAS production.
EAF-PA	1000	Significantly reduced gastric ulcers induced by acidified ethanol.

Crude methanolic extract (CME-PA); hexane fraction (HF-PA); dichloromethane fraction (DCMF-PA); ethyl acetate fraction (EAF-PA); compounds **1** (1,2,4-trihydroxy-n-heptadec-16-yne, avocadyne), **2** (1-acetoxy-2,4-dihydroxy-n-heptadec-16-ene, AcO-avocadyne), **3** (1-acetoxy-2,4-dihydroxy-n-heptadec-16-yne, AcO-avocadene), **4** (1,2,4-trihydroxy-n-heptadec-16-ene, avocadene); **M** (mixture of compounds); reduced glutathione (GSH); glutathione S-transferase (GST); catalase (CAT); malondialdehyde (MDA); myeloperoxidase (MPO); periodic acid Schiff (PAS)

All the data together allowed the identification of the compounds 1,2,4-trihydroxy-n-heptadec-16-yne (avocadyne, **1**); 1-acetoxy-2,4-dihydroxy-n-heptadec-16-ene (AcO-avocadene, **2**); 1-acetoxy-2,4-dihydroxy-n-heptadec-16-yne (AcO-avocadyne, **3**); 1,2,4-trihydroxy-n-heptadec-16-ene (avocadene, **4**); and catechin (**5**) (Fig. 1).

### Oral administration of CME-PA does not promote gastroprotection in mice

**Fig. 2:**
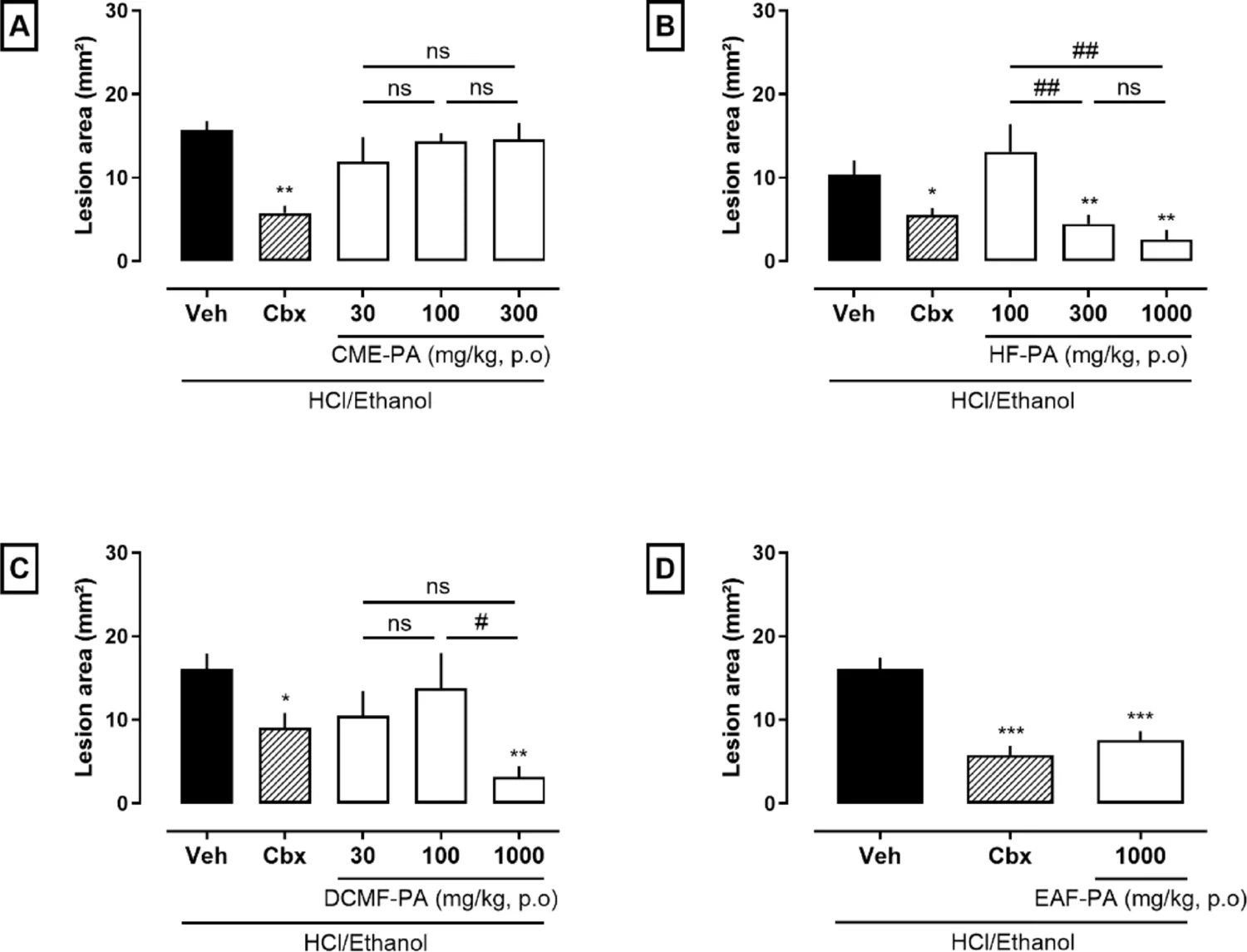
Effect of oral administration of CME-PA (Panel A) and the HF-PA (Panel B), DCMF-PA (Panel C), and EAF-PA (Panel D) fractions on acidified ethanol–induced gastric ulcers in mice. Animals were pretreated with vehicle (Veh, 1% DMSO, 1 mL/kg, p.o.), carbenoxolone (Cbx, 200 mg/kg), CME-PA (crude methanolic extract; 30, 100, or 300 mg/kg, p.o.), HF-PA (hexane fraction; 100, 300, or 1000 mg/kg, p.o.), DCMF-PA (dichloromethane fraction; 30, 100, or 1000 mg/kg, p.o.), or EAF-PA (ethyl acetate fraction; 1000 mg/kg, p.o.). Results are expressed as means ± S.E.M. (*n* = 6–8). One-way analysis of variance (ANOVA) followed by Bonferroni’s post hoc test was used. *p < 0.05, **p < 0.01, and ***p < 0.001 compared with the vehicle-treated group. ^#^*p* < 0.05 or ^##^*p* < 0.01 in comparison to the treatment group shown by the line. ns: not significant

As shown in Fig. 2A, the ulcerated vehicle group showed characteristic hemorrhagic lesions that covered an extension of 15.71 ± 1.05 mm^2^ in the gastric mucosa. However, pretreatment with CME-PA (30–300 mg/kg, p.o.) did not prevent the acidified ethanol-induced ulcers in mice. As expected, the administration of carbenoxolone, a derivative of glycyrrhetinic acid largely used as a positive control in this model, reduced the ulcer area by 63.14% (*p* = 0.0033). The macroscopic appearance of this first set of results can be seen in Supplementary Figure S6.

### Oral administration of fractions obtained from *P. americana* promotes gastroprotection at high doses in mice

As shown in Fig. 2B, oral administration of HF-PA at doses of 300 and 1000 mg/kg reduced the lesion area by 56.73% (*p* = 0.0091) and 74.93% (*p* = 0.0037), respectively, compared with the ulcerated vehicle group (10.33 ± 1.70 mm²). As expected, the administration of carbenoxolone reduced the lesion area by 46.19% (*p* = 0.0213) compared with the vehicle group.

Similarly, as illustrated in Fig. 2C and D, the DCMF-PA (1000 mg/kg) and EAF-PA (1000 mg/kg) fractions were also evaluated and produced reductions in ulcer area of 80.53% (*p* = 0.0019) and 52.91% (*p* = 0.0008), respectively, relative to the vehicle group (16.13 ± 1.80 and 16.10 ± 1.35 mm², respectively), but only at the higher dose of 1000 mg/kg. Furthermore, Fig. 2C and D show that carbenoxolone administration reduced the lesion area by 43.74% (*p* = 0.0426) and 63.98% (*p* = 0.0003), respectively, compared with the vehicle group (16.13 ± 1.80 and 16.10 ± 1.35 mm², respectively).

The macroscopic images corresponding to this set of results can be found in Supplementary Figure S6.

### Compounds **1**–**4** and the mixture (**M**) obtained from HF-PA, as well as compound **4** isolated from DCMF-PA, promote gastroprotection in mice

**Fig. 3:**
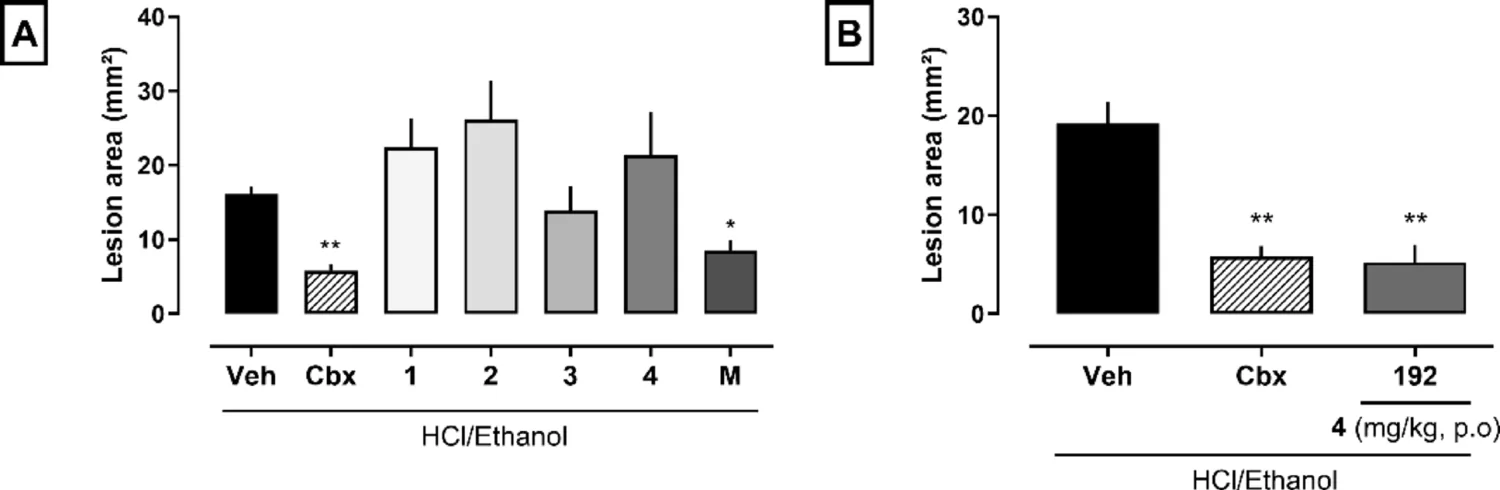
Effect of oral administration of isolated compounds **1**–**4** and a mixture of compounds (**M**) obtained from HF-PA (Panel A), and compound **4** isolated from DCMF-PA (Panel B), on acidified ethanol-induced gastric ulcers in mice. Animals were pretreated with vehicle (Veh, DMSO 1%, 1 mL/kg, p.o.), carbenoxolone (Cbx, 200 mg/kg), and compounds **1** (1,2,4-trihydroxy-n-heptadec-16-yne, avocadyne; 2.1 mg/kg, p.o.), **2** (1-acetoxy-2,4-dihydroxy-n-heptadec-16-ene, AcO-avocadene; 0.5 mg/kg, p.o.), **3** (1-acetoxy-2,4-dihydroxy-n-heptadec-16-yne, AcO-avocadyne; 1 mg/kg, p.o.), **4** (1,2,4-trihydroxy-n-heptadec-16-ene, avocadene; 7.3 mg/kg, p.o.), and **M** (mixture of compounds, 0.6 mg/kg, p.o.) obtained from the HF-PA fraction, as well as compound **4** (192 mg/kg, p.o.) isolated from DCMF-PA. Results are expressed as means ± S.E.M. (*n* = 6–8). One-way analysis of variance (ANOVA) followed by Bonferroni’s post hoc test was used. *p < 0.05 and **p < 0.01 compared with the vehicle-treated group

As shown in Fig. 3A, the compounds obtained from HF-PA (**1**–**4** and **M**) were administered at doses of 2.1, 0.5, 1, 7.3, and 0.6 mg/kg, respectively, according to their yields. Among the compounds from HF-PA, only **M** reduced the ulcer area by 47.20% (*p* = 0.0114) compared with the vehicle group (16.10 ± 1.05 mm²). As expected, the carbenoxolone produced a 63.98% (*p* = 0.0013) reduction in the lesion area compared with the vehicle group (Fig. 3A). Compounds **1**–**4**, at the tested doses, were not effective in protecting the gastric mucosa from the lesions induced by acidified ethanol.

However, compound **4** was also present in DCMF-PA in higher amounts than in HF-PA. Therefore, compound **4** was retested at a dose of 192 mg/kg, corresponding to its yield in DCMF-PA (Fig. 3B). With this dose adjustment, compound **4** produced a 73.63% (*p* = 0.0007) reduction in ulcer area compared with the vehicle group (19.17 ± 2.25 mm²). Administration of carbenoxolone, used as the positive control, reduced the occurrence of lesions by 69.75% (*p* = 0.0010) compared with the vehicle group (Fig. 3B).

The macroscopic images of the lesion areas corresponding to Fig. 3A and B can be found in Supplementary Figure S7.

**Fig. 4:**
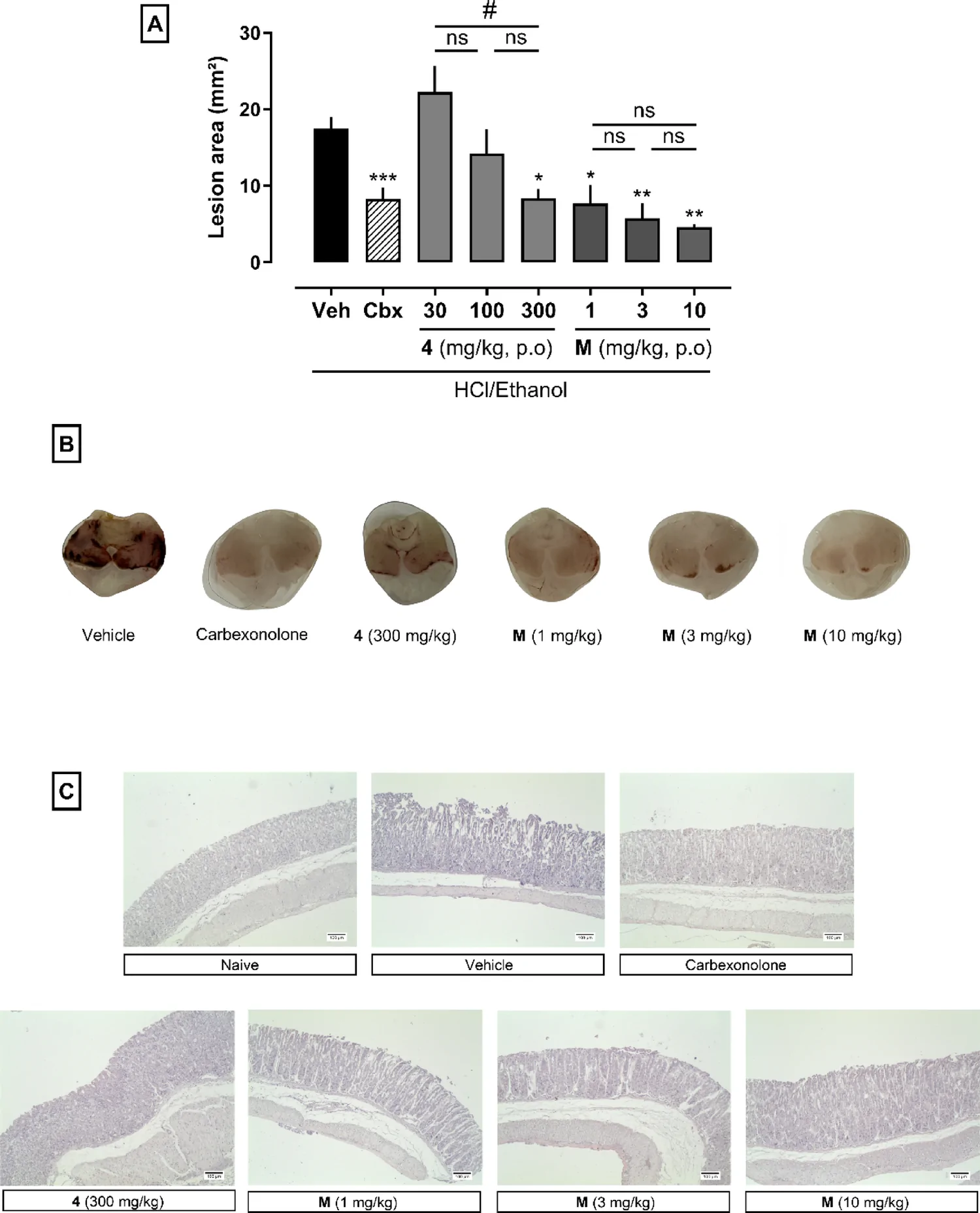
Effect of oral administration of a dose–response curve of compound **4** isolated from DCMF-PA and of a mixture of compounds (**M**) obtained from HF-PA on acidified ethanol–induced gastric ulcers in mice. Animals were pretreated with vehicle (Veh, DMSO 1%, 1 mL/kg, p.o.), carbenoxolone (Cbx, 200 mg/kg), compound **4** (1,2,4-trihydroxy-n-heptadec-16-ene, avocadene) isolated from DCMF-PA (30, 100, and 300 mg/kg, p.o.), or **M** (mixture of compounds) obtained from HF-PA (1, 3, and 10 mg/kg, p.o.). Results are expressed as means ± S.E.M. (*n* = 6–8). One-way analysis of variance (ANOVA) followed by Bonferroni’s post hoc test was used. *p < 0.05 and **p < 0.01 compared with the vehicle-treated group. ^#^*p* < 0.05 in comparison to the treatment group shown by the line. ns: not significant. Representative macroscopic (Panel A) and microscopic (Panel B) images of the gastric mucosa following acidified ethanol administration

Given that compounds **4** and mixture **M**, at doses corresponding to their yields in DCMF-PA and HF-PA, respectively, protected the gastric mucosa from acidified ethanol–induced lesions, we next evaluated their gastroprotective effects using a dose–response approach. Compound **4** was administered at doses of 30, 100, and 300 mg/kg; however, only the 300 mg/kg dose reduced the ulcerated area by 52.16% (*p* = 0.0209) compared with the vehicle (17.45 ± 1.55 mm²; Fig. 4A). In parallel, the mixture **M** was administered at doses of 1, 3, and 10 mg/kg, resulting in reductions of 56.02% (*p* = 0.0110), 66.90% (*p* = 0.0043), and 72.89% (*p* = 0.0014) in the ulcerated area, respectively, relative to the vehicle group (17.45 ± 1.55 mm²; Fig. 4A). The macroscopic and microscopic appearance of the lesions is shown in Fig. 4B and C, respectively.

### Oral administration of compound **4** and mixture **M** increases the secretion of PAS-stained mucins in the ethanol-induced gastric ulcer in mice

**Fig. 5:**
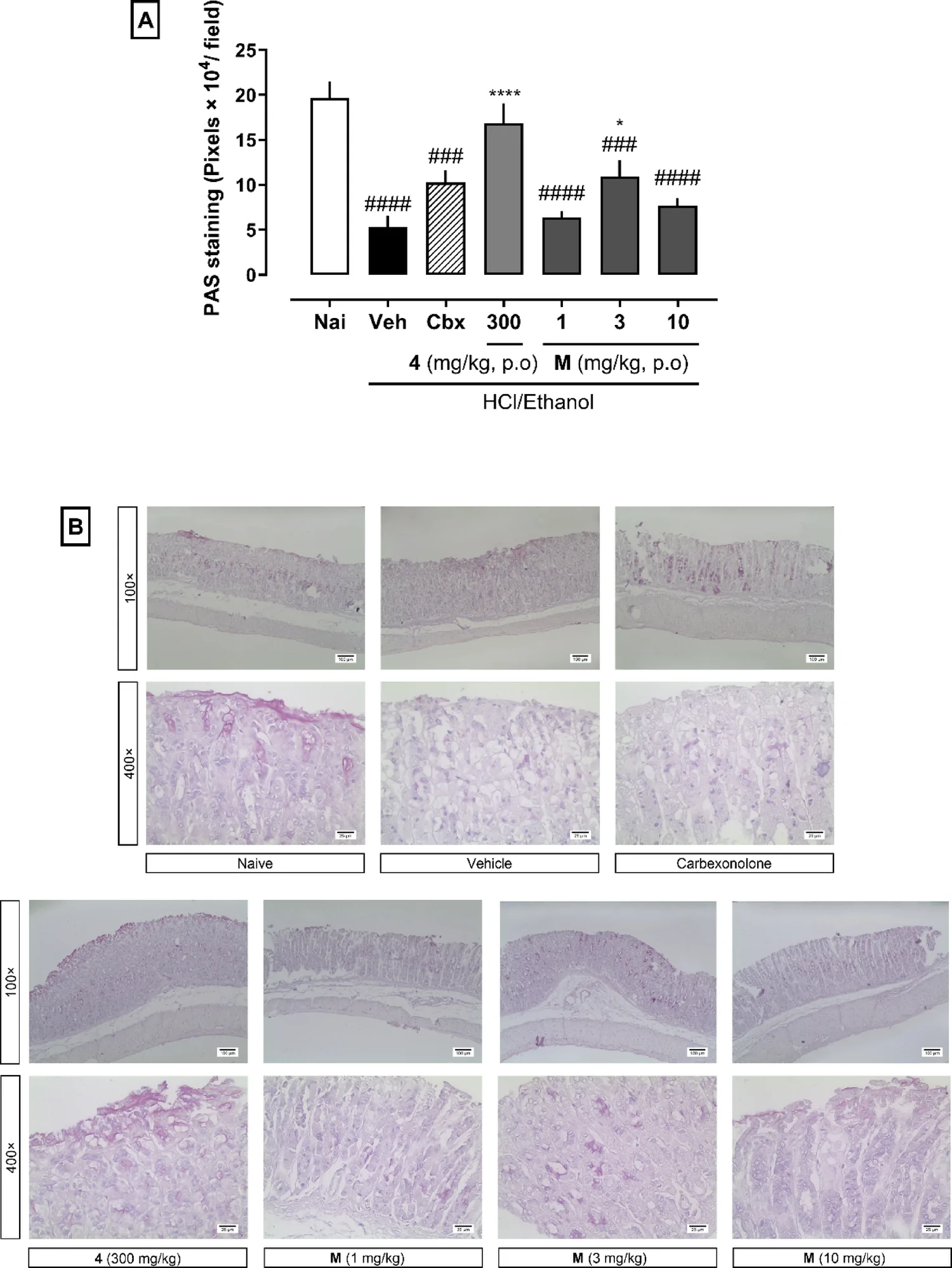
Effect of oral administration of compound **4** isolated from DCMF-PA and a mixture of compounds (**M**) obtained from HF-PA on mucin staining by the periodic acid–Schiff (PAS) method in the gastric mucosa of mice exposed to acidified ethanol. Animals were pretreated with vehicle (Veh, 1% DMSO, 1 mL/kg, p.o.), carbenoxolone (Cbx, 200 mg/kg), compound **4** (1,2,4-trihydroxy-n-heptadec-16-ene, avocadene) isolated from DCMF-PA (300 mg/kg, p.o.), or **M** (mixture of compounds) obtained from HF-PA (1, 3, and 10 mg/kg, p.o.). Results are expressed as means ± S.E.M. (*n* = 6–8). One-way analysis of variance (ANOVA) followed by Bonferroni’s post hoc test was used. ^###^*p* < 0.001 and ^####^*p* < 0.0001 compared with the naive group (Nai, non-ulcerated). *p < 0.05 and ****p < 0.0001 compared with the vehicle-treated group. Representative images showing the histochemical appearance of PAS-stained mucins (Panel B)

Figure 5A shows the effects of compound **4** (300 mg/kg) and **M** (1, 3, and 10 mg/kg) on PAS-stained mucin levels. Administration of acidified ethanol resulted in a 72.88% (*p* < 0.0001) and 47.76% (*p* = 0.0002) reduction in PAS-stained mucins in the gastric mucosa of mice treated with vehicle and carbenoxolone, respectively, compared with the naive group (19.66 ± 1.79 pixels × 10⁴/field). In contrast, only the doses of 300 mg/kg for compound **4** and 3 mg/kg for the mixture **M** increased the amount of PAS-stained mucin by 3.15- (*p* < 0.0001) and 2.04-fold (*p* = 0.0258), respectively, compared with the vehicle group (5.34 ± 1.20 pixels × 10⁴/field). However, when compared with the naive group, all tested doses of **M** (1–10 mg/kg) showed reductions of 3.06- (*p* < 0.0001), 1.79- (*p* = 0.0007), and 2.55-fold (*p* < 0.0001). Representative PAS-stained images are shown in Fig. 5B.

### Oral administration of mixture **M**, but not compound **4**, attenuates oxidative stress and inflammation in the ethanol-induced gastric ulcer in mice

**Fig. 6:**
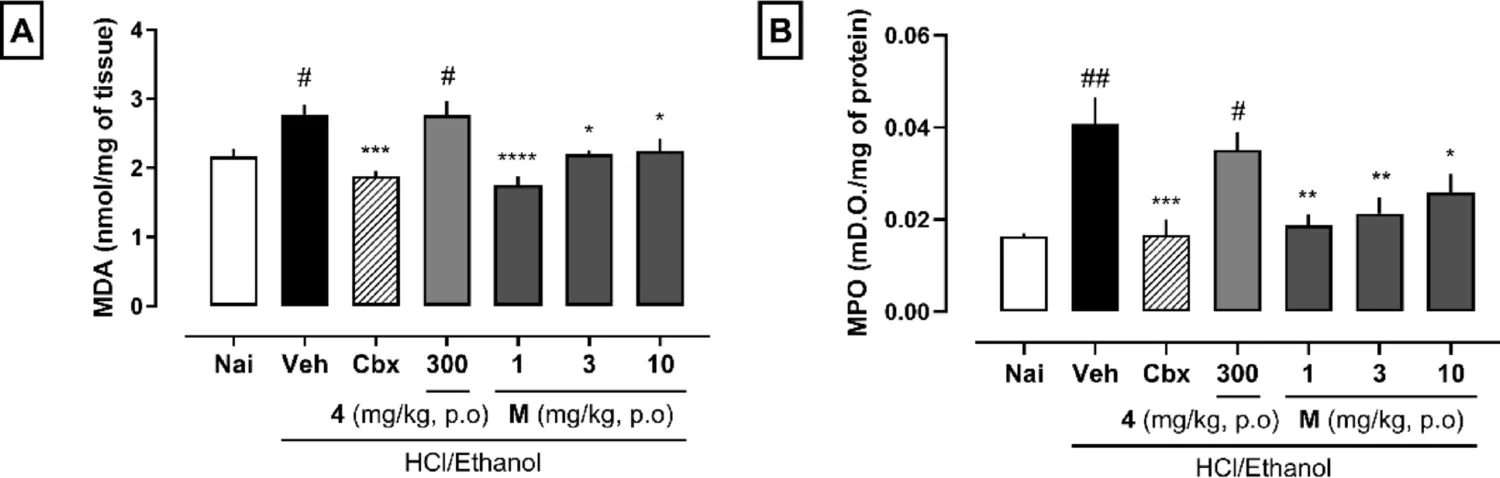
Effect of oral administration of compound **4** isolated from DCMF-PA and a mixture of compounds (**M**) obtained from HF-PA on malondialdehyde (MDA, Panel A) levels and myeloperoxidase activity (MPO, Panel B) in the gastric mucosa of mice exposed to acidified ethanol. Animals were pretreated with vehicle (Veh, 1% DMSO, 1 mL/kg, p.o.), carbenoxolone (Cbx, 200 mg/kg), compound **4** (1,2,4-trihydroxy-n-heptadec-16-ene, avocadene) isolated from DCMF-PA (300 mg/kg, p.o.), or **M** (mixture of compounds) obtained from HF-PA (1, 3, and 10 mg/kg, p.o.). Results are expressed as means ± S.E.M. (*n* = 6–8). One-way analysis of variance (ANOVA) followed by Bonferroni’s post hoc test was used. ^#^*p* < 0.05 and ^##^*p* < 0.01 compared with the naive group (Nai, non-ulcerated). *p < 0.05, **p < 0.01, ***p < 0.001, and ****p < 0.0001 compared with the vehicle-treated group

Administration of acidified ethanol induced a 28.24% (*p* = 0.0103) increase in MDA levels in vehicle-treated animals compared with the naive group (2.16 ± 0.11 nmol/mg of tissue; Fig. 6A). In contrast, treatment with carbenoxolone, as well as with mixture **M** (1, 3, and 10 mg/kg), reduced MDA levels by 32.13% (*p* = 0.0003), 36.82% (*p* < 0.0001), 20.58% (*p* = 0.0172), and 18.77% (*p* = 0.0316), respectively, relative to the vehicle group (2.77 ± 0.14 nmol/mg of tissue; Fig. 6A). However, treatment with compound **4** (300 mg/kg) did not prevent gastric mucosal lipid peroxidation, showing an increase of 27.77% (*p* = 0.0258) compared with the naive group.

Additionally, administration of acidified ethanol led to a 14.82% (*p* = 0.0094) decrease in GSH levels in vehicle-treated animals compared with the naive group (344 ± 19 µg/mg of tissue; Table 1). Similarly, the groups that received carbenoxolone or compounds **4** (300 mg/kg) and **M** (1, 3, and 10 mg/kg) also showed GSH depletion of 20.06% (*p* = 0.0007), 16.28% (*p* = 0.0068), 20.64% (*p* = 0.0008), 21.22% (*p* = 0.0003), and 22.38% (*p* = 0.0001), respectively, relative to the naive group (293 ± 04 µg/mg of tissue; Table 1).

In parallel, GST activity in the gastric mucosa of ulcerated mice treated with vehicle or with compounds **4** (300 mg/kg) and **M** (3 and 10 mg/kg) increased by 9.46- (*p* < 0.0001), 24.28- (*p* = 0.0013), 3.33- (*p* = 0.0365), and 7.64-fold (*p* = 0.0151), respectively, compared with the naive group (1.23 ± 0.09 mmol of GSH/mg of protein; Table 1). Despite this increase, treatment with compounds **4** (300 mg/kg) and **M** (1, 3, and 10 mg/kg), as well as with carbenoxolone, resulted in a reduction of GST activity by 2.57- (*p* < 0.0001), 7.30- (*p* < 0.0001), 3.50- (*p* < 0.0001), 3.17- (*p* < 0.0001), and 5.76-fold (*p* < 0.0001), respectively, when compared with the vehicle group (29.86 ± 2.52 mmol of GSH/mg of protein; Table 1).

CAT activity decreased by 44.65% (*p* = 0.0096), 71.63% (*p* < 0.0001), 61.86% (*p* = 0.0002), and 68.84% (*p* < 0.0001) in the groups treated with vehicle and with compounds **4** (300 mg/kg) and **M** (1 and 3 mg/kg), respectively, compared with the naive group (2.15 ± 2.26 mmol H_2_O_2_/mg of protein/min; Table 1). In contrast, the carbenoxolone and **M** (10 mg/kg) groups showed CAT activity like that of the naive group, with increases of 111.76% (*p* < 0.0001) and 84.03% (*p* = 0.0087), respectively, relative to the vehicle-treated group (1.19 ± 0.30 mmol H_2_O_2_/mg of protein/min; Table 1).

Finally, gastric mucosal inflammation was assessed by measuring MPO activity in animals with ulcers induced by acidified ethanol. The vehicle-treated group and the group receiving compound **4** (300 mg/kg) showed increases of 2.50- (*p* = 0.0013) and 2.18- (*p* = 0.0159) fold in MPO activity compared with the naive group (0.016 ± 0.001 mD.O/mg of protein, Fig. 5B). MPO activity was reduced by 2.35- (*p* = 0.0004), 2.10- (*p* = 0.0022), 1.90- (*p* = 0.0049), and 1.54-fold (*p* = 0.0413) in the groups treated with carbenoxolone and the mixture **M** (1, 3, and 10 mg/kg), respectively, compared with the vehicle group (0.040 ± 0.006 mD.O./mg of protein; Fig. 6B).

### CME-PA and its derived fractions showed no in vitro anti-H. pylori activity

CME-PA, as well as the HF-PA, EAF-PA fractions, and compound **4**, showed MIC values of 2000, 1000, > 4000, and 1000 µg/mL, respectively, and therefore did not exhibit in vitro anti-*H. pylori* activity. In contrast, the DCMF-PA fraction displayed moderate anti-*H. pylori* activity, with an MIC of 250 µg/mL (data not shown).

## Discussion

Looking for bioactive compounds to promote health has become a top priority for both industry and academia, and by-products are an interesting option in this search because they are a low-cost supply. Avocado seeds are a by-product that contains polyunsaturated fatty compounds and other possibly useful bioactive substances (Yahia and Woolf 2020). Thus, aiming to better understand the potential of these compounds, this study demonstrates for the first time the gastroprotective action of fractions and fatty alcohols from *P. americana* seed in acute ulcers caused by acidified ethanol, as described in Table 2. It also examines the impact of these fatty alcohols on mucosal protective factors like mucin and on the oxidative damage markers, including reduced glutathione levels, lipid peroxidation, and antioxidant enzyme activity.

Methanol is effective for extracting various compounds from plant materials because it exhibits high extraction efficiency. In addition to phenolic compounds, lipids and fatty acids were also extracted by this solvent (Lee et al. 2024; Spigno et al. 2007; Carrera et al. 2012). The gastroprotective activity of an ethyl acetate partition from *P. americana* seed extract was previously disclosed by Athaydes et al. (2019), who supported the results with their phenolic components. Since avocado seeds are known to contain a lot of fatty compounds, besides their phenolic content, the first extraction in this investigation was carried out using methanol due to its ability to extract these chemicals.

However, crude extracts often comprise a complex chemical matrix where active compounds are significantly diluted by abundant primary metabolites or where antagonistic interactions mask the biological effects (Caesar and Cech 2019). This can explain why the crude methanolic extract initially showed no gastroprotective activity. To reveal the therapeutic potential of the seed constituents, a liquid-liquid partitioning strategy was designed using solvents of increasing polarity. This fractionation successfully eliminated interfering ballast materials and concentrated on the active lipophilic and moderately polar constituents within the hexane and dichloromethane layers, thereby unmasking their gastroprotective effects and fully justifying the subsequent phytochemical isolation from these active fractions.

The goal of animal models of human diseases should be to replicate human situations as nearly as feasible. The etiology of human peptic ulcers is typically complex (Mizui and Doteuchi 1983), and for this reason several animal models have been employed to assess the antiulcer potential of substances. Among these, ethanol, acidified or not acidified, is the most used model to promote mucosal damage in rodents and is useful to investigate the gastroprotective action of compounds. The breakdown of the mucus-bicarbonate barrier, oxidative stress, and the release of pro-inflammatory cytokines are among the causes of ethanol’s ulcerogenic potential (MacMath 1990). This model was therefore adopted in this study due to its reproducibility and feasibility.

Interestingly, CME-PA could not prevent the development of gastric lesions following oral administration of acidified ethanol. Despite the negative results from CME-PA against acidified ethanol-induced ulcers, the partition of this crude extract was made to monitor the presence of some active compounds in an isolated manner, mainly those from the lipid profile found in avocado seeds. Then, the gastroprotective effect of HF-PA was confirmed at doses of 300 and 1000 mg/kg. The greater gastroprotective potency of HF-PA confirmed to us that lipid bioactive compounds can concentrate in a hexane partition due to their non-polar profile, leading to the continuation of the partition and isolation process to find these potential chemicals.

Based on our primary interest in investigating the gastroprotective effects of non-polar compounds extracted from avocado seeds, the gastroprotective effect of EAF-PA was tested only at a high single dose (1000 mg/kg) to quickly assess its potential against acidified ethanol, a result consistent with that found by Athaydes et al. (2019). Because no compounds of particular interest for this study were identified in this fraction, it made sense not to pursue further dose-response studies. However, Athaydes et al. (2019) have already proven the gastroprotective properties of the ethyl acetate fraction from avocado seeds high in phenolics against an NSAID.

Subsequently, the HF-PA fraction was first separated by OCC on silica gel using a hexane–acetone gradient, generating 94 subfractions, later pooled based on TLC profiles. These subfractions were further purified by flash chromatography, yielding fractions HF-PA **1**, **2**, **3**, and **4**. Additionally, from the HF-PA fraction, we identified a mixture of compounds (**M**), containing **2**, **3**, **4**, β-sitosterol, and other compounds (Supplementary Figure S1). Compounds **1** and **4** are PFAs, while **2** and **3** are FAEs.

There are previous reports about the antiulcer activity of β-sitosterol and its glycoside. Xiao et al. (1992) report on the anti-ulcerative activity of β-sitosterol glycoside and its aglycone in rats using acetic acid and cold stress models, the effect being greater with the aglycone. Furthermore, Navarrete et al. (2002) reported gastroprotective activity of β-sitosterol and sitosterol-3-O-β-glucoside in rats ulcerated by ethanol, aspirin, and histamine, noting that the sitosterol-glycoside in this report was more effective than sitosterol.

Therefore, given the presence of β-sitosterol in **M** and the fact that avocado is a rich source of health-promoting lipophilic compounds (Dreher and Davenport 2013), we evaluated the gastric ulcer-protective effects of these four isolated compounds, as well as of the mixture **M** obtained from HF-PA. The tested doses of compounds **1**, **2**, **3**, **4**, and **M** obtained from HF-PA were 2.1, 0.5, 1, 7.3, and 0.6 mg/kg, respectively, and were calculated in accordance with their yield and the minimum gastroprotective dose of HF-PA, an approach used in bio-guided pharmacological studies (De Barros et al. 2016) that reduces animal use because it drives the testing of a unique dose for each compound in addition to highlighting the role of each compound in the achieved effect of the original fraction. However, it is important to note that this yield-based approach limits direct comparisons of intrinsic potency among the compounds, as their individual exposure levels varied significantly. Therefore, while this bio-guided approach aligns with ethical principles to reduce animal use, it prevents us from ruling out the gastroprotective potential of compounds **1**–**3** at higher doses. Therefore, the lack of activity observed for these compounds should not be interpreted as absolute biological inactivity, but rather as an absence of effect at specific low doses tested. Future systematic dose-response studies are required to determine the true intrinsic potency of each isolated compound.

Of the compounds obtained from HF-PA, only the mixture of compounds (**M**) at the specific doses evaluated showed gastroprotective activity, which was subsequently tested in a dose-response curve (1, 3, and 10 mg/kg) and demonstrated a gastroprotective effect in all tested doses. When tested separately, the compounds from HF-PA did not present significant effects, suggesting a potential additive or combinatorial effect occurring between the compounds, which may account for the gastroprotective activity observed in the mixture (**M**). These findings indicate that the combination of phytochemicals present in mixture **M** may potentiate their biological effects through complementary mechanisms of action. Indeed, the gastroprotective effect promoted by **M** was accompanied by the reduction of oxidative damage, attenuation of gastric mucosal inflammation, and increased mucin production, suggesting the simultaneous modulation of multiple protective pathways. Similar effects have been reported for mixtures of plant extracts, which exhibited superior gastroprotective efficacy compared to isolated compounds, likely due to complex interactions and cooperation among their bioactive constituents (Wang et al. 2015; Halim et al. 2017; Park et al. 2019), which can be assessed in future studies.

DCMF-PA promoted gastroprotection at a high dose, 1000 mg/kg; however, compound **4** was also isolated from this fraction and, in a dose corresponding to its yield in DCMF-PA (192 mg/kg), proved effective in reducing lesion formation following acidified ethanol administration. It is important to note that when compound **4** was tested at 7.3 mg/kg (a dose reflecting its yield in HF-PA), it showed no gastroprotective effect, which could easily be misinterpreted as biological inactivity. However, when evaluated at 192 mg/kg (reflecting its higher yield in DCMF-PA), compound **4** demonstrated significant gastroprotective activity. This discrepancy reinforces that the single-dose negative results obtained for compounds **1–3** may simply reflect insufficient dose exposure rather than a true lack of pharmacological activity.

Subsequently, avocadene **(4)** was evaluated in a dose–response curve (30, 100, and 300 mg/kg) and exhibited gastroprotective activity only at the 300 mg/kg dose, which is 300-fold higher than the minimum effective dose of mixture **M** required to exert gastroprotection. Furthermore, it is relevant to remember that compound **4** was evaluated at 192 mg/kg because this dose represents the exact mass equivalent yielded from 1000 mg/kg of the DCMF-PA fraction. The significant gastroprotective effect observed at 192 mg/kg, and its subsequent absence at the lower dose of 100 mg/kg in the dose-response curve, supports that compound **4** is a primary active principle driving the fraction’s efficacy. However, we cannot rule out that the overall activity of HF-PA and DCMF-PA may also rely on the contribution of other minor constituents. Phytocomplexes often exhibit enhanced efficacy due to additive or synergistic interactions among multiple compounds, and further studies would be valuable to fully map these interactions. Moreover, the gastroprotective effect of compound **4** was associated with an increase in mucin production but did not prevent the elevation of oxidative stress or inflammation in the gastric mucosa.

The gastroprotective action of avocadene (**4**) described here reinforces the potential value of this substance to treat gastric diseases, given that its anti-*H. pylori* efficacy was recently described (Le et al. 2025). However, for translational purposes, it would be optimal if the minimal effective dose of **4** for gastroprotection were reduced; nevertheless, its pharmacokinetics can be improved using pharmaceutical technology systems, lowering the amount necessary for biological action. Indeed, avocadene’s efficacy and bioavailability were increased in in vitro experiments (Ahmed et al. 2020) utilizing a self-emulsifying drug delivery system that spontaneously creates oil-in-water (O/W) emulsions. This method was utilized to encapsulate commercially available pure crystals of AvoB (a mixture containing avocadene and avocadyne), as previously described (Ahmed et al. 2020). Thus, future research may address the dosage and efficacy of this compound using such self-emulsifying systems. As previously described, avocadene (**4)** is one of the constituents of the mixture (**M**), and these findings further support the probable, but not yet proven, hypothesis that the combined action of the constituents in **M** is necessary to achieve effective gastric mucosal preservation at lower doses. A significant issue to consider is that some chemicals present in **M** may self-assemble at the O/W emulsion interface, influencing bioavailability and the dose necessary for the effect. In addition, future studies can extract larger quantities using dichloromethane and investigate their antiulcer efficacy using more complex models, such as acetic-acid-induced chronic ulcers, which require prolonged treatment and therefore a larger quantity of substances.

In relation to the antimicrobial assay, only the DCMF-PA fraction demonstrated moderate in vitro anti-*H. pylori* activity (MIC = 250 µg/mL). This indicates that the principal evidence of this study concerns gastroprotection rather than a direct antimicrobial effect. Although the other materials were inactive under the tested conditions, this negative result does not completely rule out their anti-*H. pylori* potential in different experimental models. Therefore, the activity of DCMF-PA serves as a complementary finding, while gastric tissue protection remains the primary focus of this work.

Regarding safety, no mortality or treatment-related clinical signs of acute toxicity (such as lethargy, abnormal gait, or respiratory changes) were observed during the experimental period, even at the highest doses administered (1000 mg/kg for the DCMF-PA or EAF-PA and 300 mg/kg for compound **4**) (data not shown). These preliminary observations suggest a favorable acute safety profile; however, formal preclinical toxicological studies are still required to fully establish their safety parameters.

The gastric mucus layer constitutes an essential physical barrier that protects the gastric mucosa from direct contact with gastric acid and pepsin, while also hindering the penetration of injurious agents due to its viscoelastic properties (Allen and Flemström 2005; Nordman et al. 1998). However, ethanol is known to penetrate gastric mucosa and solubilize the protective mucus layer, thereby exposing the underlying tissue to the harmful actions of gastric acid and pepsin and rendering the mucosa more susceptible to gastric ulcer formation (Wong et al. 1986). In this context, the enhanced PAS staining observed in rats treated with the mixture **M** (3 mg/kg) and compound **4** (300 mg/kg) indicates a strengthening of this key defensive barrier, thereby contributing to the observed gastroprotective effect.

In addition to disrupting the mucus layer, ethanol also increases oxidative stress in the gastric mucosa, leading to enhanced production of reactive oxygen species (ROS), lipid peroxidation, depletion of antioxidant defenses, and elevated levels of pro-inflammatory cytokines (Navvabi et al. 2025). Glutathione (GSH) is a tripeptide with high antioxidant capacity that neutralizes ROS by donating electrons from its sulfhydryl group (Averill-Bates 2023), and reduced levels of this molecule have been implicated in the pathogenesis of gastric ulcers (Gokce and Dag 2017).

Interestingly, the depletion of GSH in the ulcerated-vehicle group was accompanied by a greater increase in GST activity, Interestingly, the depletion of GSH in the ulcerated-vehicle group was accompanied by an increase in GST activity. While this tripeptide serves as a substrate for GST (Fraternale et al. 2009), our current data do not establish a direct causal link between enhanced GST activity and GSH depletion. However, despite the gastroprotective effects observed, treatments with the mixture **M** and 4 did not prevent GSH depletion in the gastric mucosa. It is also important to note that in groups receiving mixture **M** (3 and 10 mg/kg) or compound **4** (300 mg/kg), the GST activity was also enhanced, which could hypothetically be associated with the reduction in GSH levels, although this mechanism requires further experimental validation. Curiously, mice treated with **M** at 1 mg/kg exhibited reduced levels of GSH concurrent with reduced levels of GST activity; in this case, it could be related that other unmeasured pathways, such as glutathione peroxidase (GPx) activity or accumulation of oxidized reactive substrates, might play a role in the depletion of this antioxidant tripeptide. Nevertheless, these proposed mechanisms remain strictly hypothetical since GPx activity was not experimentally evaluated in the present study.

Moreover, the activity of catalase (CAT), an enzyme that catalyzes the decomposition of hydrogen peroxide into water and oxygen (Chelikani et al. 2004), was diminished in the ulcerated group, potentially elucidating the elevated levels of MDA, a recognized biomarker of lipid peroxidation (Tsikas 2017), in this group. Conversely, the pre-treatment with **M** at 10 mg/kg normalized the CAT activity to levels like the non-ulcerated group (naive group), which was reflected in the reduced MDA levels in this group. However, ulcerated mice pretreated with **M** at doses of 1 or 3 mg/kg exhibited a decrease in gastric MDA levels, despite diminishing CAT activity in these groups. In discussing these findings, it is crucial to note that, alongside CAT, the GPx enzyme serves as an alternate mechanism for the reduction of peroxides, hence preventing lipid peroxidation, and that this enzyme necessitates GSH to facilitate this reaction. Thus, in the mice pretreated with M at lower dosages, the maintenance of lowered MDA levels may have come at the expense of GPx activity, resulting in lower GSH levels despite no increase in GST activity, particularly at the 1 mg/kg dose. In addition, β-sitosterol, one of the phytoconstituents of mixture **M**, has well-documented antioxidant properties in the literature (Baskar et al. 2012; Zhang et al. 2023; Wu et al. 2025).

MPO is an enzyme stored in the azurophilic granules of neutrophils and is closely associated with the progression of several pathological conditions due to its ability to amplify inflammation and oxidative stress, ultimately leading to tissue damage (Ray and Katyal 2016). Treatment with the mixture **M** reduced MPO activity in gastric tissue at 1, 3, and 10 mg/kg, indicating that this combination of compounds can attenuate ethanol-induced inflammatory injury and contributes to its cytoprotective effects on the gastric mucosa. In contrast, although the isolated compound **4** was able to protect gastric mucosa against lesion formation, it did not reduce MPO activity or MDA levels in gastric tissue. These findings suggest that modulation of neutrophil infiltration and lipid peroxidation are not central mechanisms underlying the gastroprotective effect of the isolated compound, further supporting the hypothesis that the anti-inflammatory and antioxidant actions observed with the mixture arise from synergistic interactions among its phytochemical constituents. Although the observed reduction in MPO activity suggests an anti-inflammatory component to the gastroprotective effect, further investigation into specific inflammatory pathways and mediators in future studies remains awaited, including the measurement of cytokines such as TNF or IL-1β.

## Limitations and future directions

Although this study provides valuable insights into the anti-ulcer potential of the fatty compounds from *Persea americana* seed, several limitations must be acknowledged. First, our findings are based on an acute ulcer model, and the effects have not yet been validated in chronic ulcer-healing experiments, which better replicate long-term clinical conditions; future studies can address this issue using a chronic ulcer model, such as the acetic acid-induced gastric ulcer.

Additionally, a limited dose-response characterization was performed for several tested compounds, and a rigorous synergy experiment is still required to fully understand their combined effects. Furthermore, the characterization of the mixture **M** remains incomplete, and pharmacokinetic data are currently lacking, limiting our understanding of the compounds’ absorption, distribution, and metabolism profiles. Finally, the study relied on limited mechanistic measurements, which restrict a deeper exploration of the underlying molecular pathways, and future research can explore this in complementary and sequential studies.

Despite these limitations, this work establishes a solid foundation and justifies further investigations. Future studies will focus on expanding the mechanistic assays, performing chronic ulcer models, and gathering pharmacokinetic data to fully elucidate the therapeutic and synergistic potential of these compounds.

## Conclusions

This study identified four compounds from the hexane fraction of *P. americana* seeds: avocadyne (**1**), AcO-avocadene (**2**), AcO-avocadyne (**3**), and avocadene (**4**), with compound **4** also isolated from the DCMF-PA fraction. A mixture (**M**) containing compounds **2**, **3**, and **4**, along with β-sitosterol and other compounds, showed gastroprotective activity against ethanol-induced ulcers at lower doses, suggesting synergistic interactions. Compound **4** alone demonstrated gastroprotection only at doses > 192 mg/kg but didn’t enhance antioxidant defenses. The mixture’s effect may involve enhancing antioxidant defenses, reducing inflammation, and increasing mucin production. Given *P. americana* seeds are typically discarded, these findings highlight their potential as a natural resource for preventing peptic ulcer disease. Future research is advised to examine the gastroprotective properties of fractions and isolated compounds from *P. americana* seeds utilizing alternative gastric ulcer models, including NSAID- or stress-induced ulcers, and to assess their ulcer-healing capabilities through acetic acid-induced chronic ulcers, alongside further studies to clarify the mechanisms underlying their effects.

## Supplementary Information

Below is the link to the electronic supplementary material.

Supplementary Material 1

## Data Availability

No datasets were generated or analysed during the current study.
