# Supplementary material for: Gastroprotective potential of fatty compounds from Persea americana seed: an experimental report
Source: Inflammopharmacology. 2026 Oct 8;34(11):7303–19. doi: 10.1007/s10787-026-02419-8 (PMC13768362; doi:10.1007/s10787-026-02419-8)
Supplement: Supplementary file 1 [file 10787_2026_2419_MOESM1_ESM.docx]

**Gastroprotective potential of fatty compounds from** *Persea americana* **seed: an experimental report**

Greice Rafaele Alves^a^, Benhur Judah Cury^d^, Bruna Longo^d^, Alexandre Bella Cruz^a,c^, Raphael Gilioli Heineck^b^, Rafaela Carvalho Faustino^b^, Yasmim Damian Pompeo Benevides^a^, Luisa Mota da Silva^d^, and Rivaldo Niero^a,c^*.

*^a^Postgraduate Program in Pharmaceutical Sciences, ^b^Multiuser Analytical Instrumentation Laboratory, and ^c^Chemical Pharmaceutical Research Center (NIQFAR), University of Vale do Itajaí (UNIVALI), Rua Uruguai 458, Itajaí, SC, Brazil, 88.302.901. ^d^Department of Pharmacology, Federal University of Santa Catarina, Florianópolis, Santa Catarina, Brazil, 88.040.900.*

* **Corresponding author:**

Tel.: +55 47 99931-9431

e-mail: lu.isamota@hotmail.com

**Supplementary material**

**Figure S1.** TIC–ESI-MS chromatographic profile of mixture of compounds (**M**) (Panel A) from *P. americana* seeds. ESI-MS product ion spectra of compounds **2** (Panel B), **3** (Panel C), **4** (Panel D), and β-sitosterol (Panel E).

**Figure S2**. NMR spectrum ^1^H (Panel A) and ^13^C (Panel B) of compound **1** in CDCl_3_ using TMS as reference standard. DI-MS fragmentation (Panel C) and FTIR-ATR (Panel D).

**Figure S3**. NMR spectrum ^1^H (Panel A) and ^13^C (Panel B) of compound **2** in CDCl_3_ using TMS as reference standard. DI-MS fragmentation (Panel C) and FTIR-ATR (Panel D).

**Figure S4.** NMR spectrum ^1^H (Panel A) and ^13^C (Panel B) of compound **3** in CDCl_3_ using TMS as reference standard. DI-MS fragmentation (Panel C) and FTIR-ATR (Panel D).

**Figure S5**. NMR spectrum ^1^H (Panel A) and ^13^C (Panel B) of compound **4** in CDCl_3_ using TMS as reference standard. DI-MS fragmentation (Panel C) and FTIR-ATR (Panel D).

**Figure S6.** Representative macroscopic images illustrating the effects of oral administration of CME-PA (Panel A), HF-PA (Panel B), DCMF-PA (Panel C), and EAF-PA (Panel D) fractions on acidified ethanol–induced gastric ulcers in mice. Animals were pretreated with vehicle (1% DMSO, 1 mL/kg, p.o.), carbenoxolone (200 mg/kg), CME-PA (30, 100, or 300 mg/kg, p.o.), HF-PA (100, 300, or 1000 mg/kg, p.o.), DCMF-PA (30, 100, or 1000 mg/kg, p.o.), or EAF-PA (1000 mg/kg, p.o.).

**Figure S7.** Representative macroscopic images illustrating the effects of oral administration of isolated compounds **1**–**4** and a mixture (**M**) obtained from HF-PA (Panel A) and compound **4** isolated from DCMF-PA (Panel B) on acidified ethanol–induced gastric ulcers in mice. Animals were pretreated with vehicle (DMSO 1%, 1 mL/kg, p.o.), carbenoxolone (200 mg/kg), and compounds **1** (2.1 mg/kg, p.o.), **2** (0.5 mg/kg, p.o.), **3** (1 mg/kg, p.o.), **4** (7.3 mg/kg, p.o.), and mixture **M** (0.6 mg/kg, p.o.) obtained from the HF-PA fraction, as well as compound **4** (192 mg/kg, p.o.) isolated from DCMF-PA.

**Table 1.** Comparative ¹H and ¹³C NMR spectroscopic data for compounds **1** and **4** (in CDCl₃, δ in ppm)

|  | Values in the literature * | | Compound 1 | | Values in the literature * | | Compound 4 | |
| --- | --- | --- | --- | --- | --- | --- | --- | --- |
|  | **¹³C NMR** | **¹H NMR** | **¹³C NMR** | **¹H NMR** | **¹³C NMR** | **¹H NMR** | **¹³C NMR** | **¹H NMR** |
| 1 | 66.80 | 3.49 (dd, J = 11; 6.5) | 66.78 | 3.48 | 66.82 | 3.49 (dd, J = 11; 6.5) | 66.78 | 3.49 (dd, J = 11.1; 6.0) |
| 1' | - | 3.65 (dd, J = 11; 3) | - | 3.65 | - | 3.65 (dd, J = 11; 3) | - | 3.65 (dd, J = 11.1; 3.3) |
| 2 | 72.66 | 3.98 m | 72.67 | 3.96 | 72.62 | 3.98 m | 72.69 | 3.96 m |
| 3 | 38.94 | 1.57 m | 38.91 | 1.56 | 38.99 | 1.58 m | 38.91 | 1.56 m |
| 4 | 72.46 | 3.91 m | 72.41 | 3.88 | 72.55 | 3.91 m | 72.35 | 3.87 m |
| 5 | 38.28 | 1.42 m | 38.24 | 1.47 | 38.34 | 1.48 m | 38.23 | 1.47 m |
| 6–14 | 29.55; 29.48; 29.09; 28.48; 25.31 | 1.26–1.43 m | 29.56; 29.47; 29.09; 28.48; 25.33 | 1.26 | 29.59; 29.50; 29.16; 28.95; 25.31 | 1.44–1.26 m | 29.61; 29.51; 29.16; 28.95; 25.37 | 1.26 (Strong signal) |
| 15 | 18.39 | 2.18 (td, J = 7; 2.5) | 18.39 | 2.15 (td, J = 6.9; 2.4) | 33.83 | 2.04 m | 33.82 | 2.02 m |
| 16 | 84.82 | - | 84.82 | - | 139.27 | 5.82 (ddt, J = 17; 10; 6.5) | 139.27 | 5.82 (ddt, J = 17.1; 10.5; 6.6) |
| 17 cis | 68.03 | 1.94 (t, J = 2.5) | 68.05 | 1.94 (t, J = 2.7) | 114.09 | 4.93 (ddt, J = 10; 2; 1.5) | 114.09 | 4.93 (dd J = 13.8; 2.1) |
| 17 trans |  |  |  |  | - | 4.99 (dd, J = 17; 2; 1.5) | - | 4.96 (dd, J = 17.1; 1.5) |

**^*^**Oberlies et al. (1998).

**Table 2.** Comparative ¹H and ¹³C NMR spectroscopic data for compounds **2** and **3** (in CDCl₃, δ in ppm)

|  | Values in the literature * | | Compound 2 | | Values in the literature * | | Compound 3 | |
| --- | --- | --- | --- | --- | --- | --- | --- | --- |
|  | **¹³C NMR** | **¹H NMR** | **¹³C NMR** | **¹H NMR** | **¹³C NMR** | **¹H NMR** | **¹³C NMR** | **¹H NMR** |
| 1 | 68.51 | 4.11 (m) 4.00 (m) | 68.55 | 4.10 (m) 4.02 (m) | 68.82 | 4.09 (m) 3.97 (m) | 68.54 | 4.07 (m) 3.98 (m) |
| 2 | 70.75 | 4.11 (m) | 70.79 | 4.10 (m) | 71.08 | 4.09 (m) | 70.78 | 4.09 (m) |
| 3 | 38.98 | 1.60 (m) | 39.02 | 1.60 (m) | 39.33 | 1.56 (m) | 39.01 | 1.56 (m) |
| 4 | 72.49 | 3.89 (m) | 72.52 | 3.98 (m) | 72.79 | 3.87 (m) | 72.50 | 3.88 (m) |
| 5 | 38.18 | 1.47 (m) | 38.18 | 1.49 (m) | 38.49 | 1.44 (m) | 38,16 | 1.47 (m) |
| 6 | 25.34 | 1.27 (m) | 25.30 | 1.25 (m) | 25.63 | 1.24 (m) | 25.28 | 1.26 (m) |
| 7 | 28.98 | 1.27 (m) | 28.94 | 1.25 (m) | 29.90 | 1.24 (m) | 29.55 | 1.26 (m) |
| 8 | 29.18 | 1.27 (m) | 29.15 | 1.25 (m) | 29.89 | 1.24 (m) | 29.47 | 1.26 (m) |
| 9 | 29.54 | 1.27 (m) | 29.50 | 1.25 (m) | 29.89 | 1.24 (m) | 29.47 | 1.26 (m) |
| 10 | 29.62 | 1.27 (m) | 29.58 | 1.25 (m) | 29.87 | 1.24 (m) | 29.41 | 1.26 (m) |
| 11 | 29.62 | 1.27 (m) | 29.58 | 1.25 (m) | 29.81 | 1.24 (m) | 29.01 | 1.26 (m) |
| 12 | 29.62 | 1.27 (m) | 29.58 | 1.25 (m) | 29.43 | 1.24 (m) | 28.92 | 1.26 (m) |
| 13 | 29.54 | 1.27 (m) | 29.50 | 1.25 (m) | 29.08 | 1.48 (m) | 28.75 | 1.49 (m) |
| 14 | 29.18 | 1.37 (m) | 29.15 | 1.46 (m) | 28.82 | 1.36 (m) | 28.48 | 1.37 (m) |
| 15 | 33.85 | 2.04 br q (J = 7) | 33.82 | 2.02 (J = 9) | 18.75 | 2.15 dt (J = 7.3; 2.7) | 18.39 | 2.15 (dt, J = 6.9; 2.7) |
| 16 | 139.14 | 5.82 ddt (J = 17; 10.5; 6.7) | 139.14 | 5.82 (dd, 16.8; 10.2) | 68.31 | - | 68.04 | - |
| 17 | 114.01 | 4.93 ddt (J = 10.5; 2.1; 1.2) 4.99 ddt (J = 17; 1.6; 1.2) | 114.09 | 4.93 (ddt, 10.2; 2.1; 1.2) 4.96 (ddt, 17.4; 2.1; 1.8) | 85.06 | 1.91 t (J = 2.7) | 84.82 | 1.92 (t, J = 2.7) |
| Ca | 171.09 | - | 171.23 | - | 171.35 | - | 171.24 | - |
| Cb | 20.96 | 2.09 s | 20.89 | 2.08 | 21.45 | 2.08 s | 20.89 | 2.08 s |
| OH | - | 2.96 br s | - | 2.52 | - | 2.63 br s | - | 2.51 br s |
| OH | - | 3.58 br s | - | 3.66 br s | - | 3.44 br s | - | 3.62 br s |

**^*^**Domergue et al*.* (2000).
